# Mesenchymal stem cell secreted vesicles provide novel opportunities in (stem) cell-free therapy

**DOI:** 10.3389/fphys.2012.00359

**Published:** 2012-09-06

**Authors:** Serena Rubina Baglio, D. Michiel Pegtel, Nicola Baldini

**Affiliations:** ^1^Laboratory for Orthopaedic Pathophysiology and Regenerative Medicine, Istituto Ortopedico RizzoliBologna, Italy; ^2^Department of Pathology, Cancer Center Amsterdam, VU University Medical CenterAmsterdam, Netherlands

**Keywords:** mesenchymal stem cell (MSC), microvesicles, exosomes, regenerative medicine, therapy

## Abstract

Mesenchymal stem cells (MSCs) are adult multipotent cells that give rise to various cell types of the mesodermal germ layer. MSCs are of great interest in the field of regenerative medicine and cancer therapy because of their unique ability to home to damaged and cancerous tissue. These cells also regulate the immune response and contribute to reparative processes in different pathological conditions, including musculoskeletal and cardiovascular diseases. The use of MSCs for tissue repair was initially based on the hypothesis that these cells home to and differentiate within the injured tissue into specialized cells. However, it now appears that only a small proportion of transplanted MSCs actually integrate and survive in host tissues. Thus, the predominant mechanism by which MSCs participate in tissue repair seems to be related to their paracrine activity. Indeed, MSCs provide the microenvironment with a multitude of trophic and survival signals including growth factors and cytokines. Recent discoveries suggest that lipid microvesicles released by MSCs may also be important in the physiological function of these cells. Over the past few years the biological relevance of micro- and nano-vesicles released by cells in intercellular communication has been established. Alongside the conventional mediators of cell secretome, these sophisticated nanovesicles transfer proteins, lipids and, most importantly, various forms of RNAs to neighboring cells, thereby mediating a variety of biological responses. The physiological role of MSC-derived vesicles (MSC-MVs) is currently not well understood. Nevertheless, encouraging results indicate that MSC-MVs have similar protective and reparative properties as their cellular counterparts in tissue repair and possibly anti-cancer therapy. Thus, MSC-MVs represent a promising opportunity to develop novel cell-free therapy approaches that might overcome the obstacles and risks associated with the use of native or engineered stem cells.

## The therapeutic potential of mesenchymal stem cells

Over the last decades, adult stem cells have been extensively studied with regard to their potential implications in regenerative medicine. The multipotent precursors of the bone marrow stroma were the first adult stem cells to be identified (Till and McCulloch, 1964; Friedenstein et al., 1970) and are still now a focus of great interest because of their ability to home to damaged sites, function in tissue repair and regeneration and modulate the immune response. As a result of their self-renewal potential and of their ability to differentiate to various phenotypes of the mesenchymal germ layer, these non-hematopoietic stromal cells are currently referred to as mesenchymal stem cells (MSCs) (Caplan, 1991).

In the bone marrow MSCs represent about the 0.01% of the mononuclear cells and provide the structural and functional support for hematopoietic stem cells (HSCs) in their niche (Johnson and Dorshkind, 1986; Pittenger et al., 1999). However, MSCs have been isolated from a variety of fetal and adult tissues including placenta, umbilical cord blood, adipose tissue (Lee et al., 2004), skeletal muscle, peripheral blood (Bosch et al., 2000; Zvaifler et al., 2000), dental pulp, and, more recently, endometrium and menstrual blood (Musina et al., 2008). Among the various sources, adipose tissue is gaining more and more interest because adipose-derived MSC are available in large amounts from liposuction procedures and thus considered major candidates for future regenerative medicine approaches (Schreml et al., 2009).

### MSC characteristics

The identification and the characterization of MSCs have been widely discussed elsewhere (Dominici et al., 2006). The absence of known specific MSC-restricted markers and the observation that the morphology of these cells can vary from spindle to trapezoid shape depending on culture conditions and passage, render it challenging to univocally identify MSCs. For this reason, the International Society for Cellular Therapy (ISCT) established minimal requirements to designate MSCs, i.e., (1) plastic adherence, (2) expression of CD73, CD90 and CD105, and negativity for various hematopoietic markers, and (3) ability to differentiate into mesenchymal cell types including adipocytes, chondrocytes and osteoblasts (Dominici et al., 2006).

In spite of these efforts, there is still a high need to further characterize the biology of these adult stem cells. In particular, plastic adherence does not appear an essential characteristic of MSCs, as conceived previously. Recent studies from multiple laboratories have shown the existence of non-adherent MSC (NA-MSC) subpopulations that display the same multipotent potential of adherent MSCs. Moreover, the non-adherent MSCs present the same ability to migrate to damaged tissues *in vivo* as adherent MSCs and also function in tissue repair and regeneration (Leonardi et al., 2009; Zhang et al., 2009).

The surface antigen pattern is also an aspect of MSC characterization to be carefully considered because the expression of markers changes depending on the surrounding environment, during culture and upon exogenous stimuli (Dominici et al., 2006).

Finally, concerning the multipotent potential of these cells, the existence of a subpopulation within bone marrow-derived MSCs capable of differentiating not only into the same mesodermal-lineage, but also into other lineages of the ectodermal and endodermal germ layers has been proposed, but is still strongly debated (Dezawa et al., 2004, 2005; Trzaska et al., 2007; Snykers et al., 2011).

**Figure 1:**
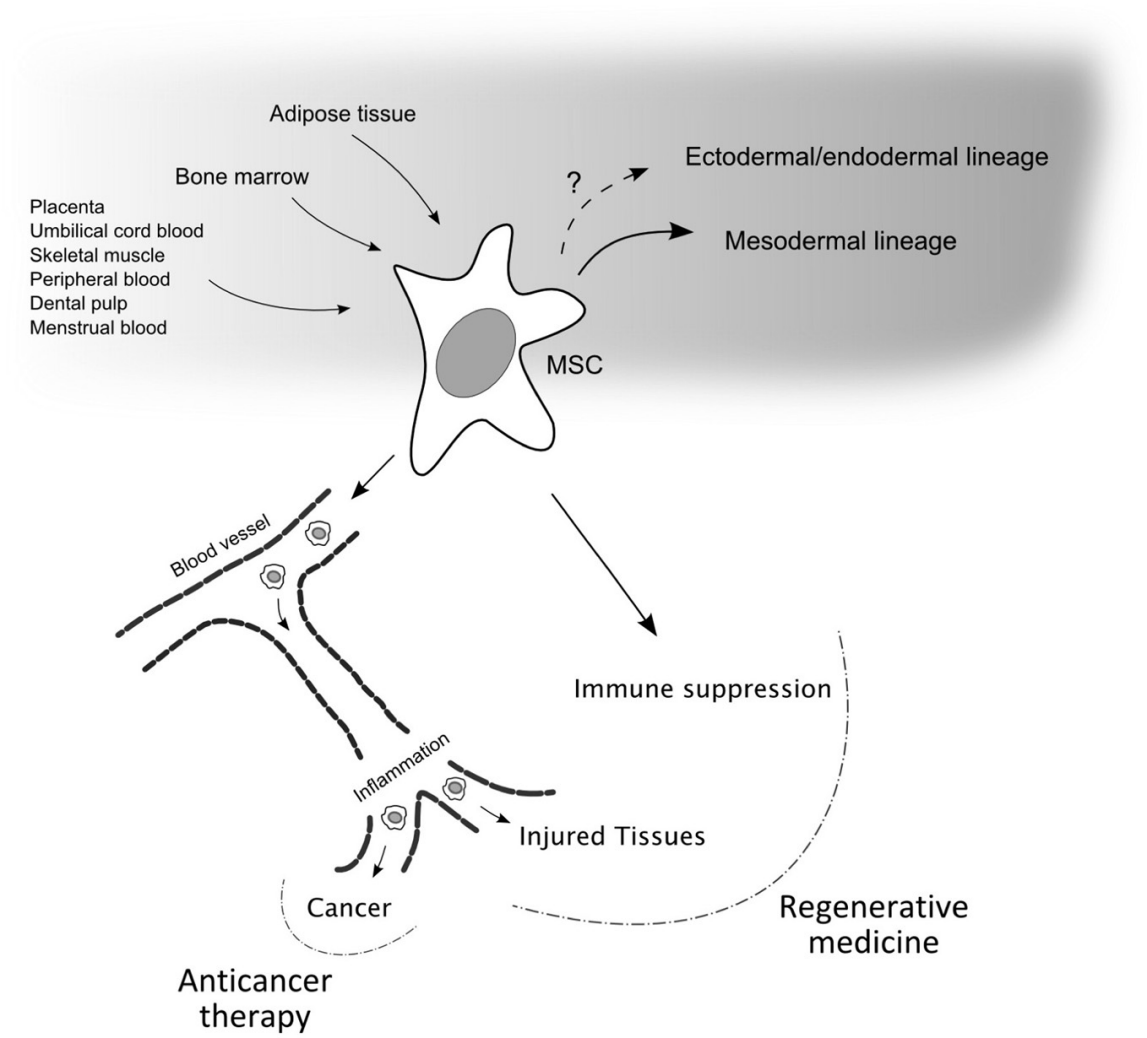
**Mesenchymal stem cell properties and relevance for therapeutic applications.** Mesenchymal stem cells (MSCs) can be isolated from different sources, in particular from bone marrow and adipose tissues, and can differentiate into various cell types of the mesodermal germ layer. The possibility that these adult stem cells can differentiate in cells of the other germ layers is not excluded and is still debated. Two main properties make MSCs good candidates for therapeutic applications. First, their ability to enter the blood circulation and home to sites of inflammation, i.e., damaged and cancerous tissues, where MSCs can release a multitude of trophic factors. Second, MSCs have the ability to suppress the immune system via different mechanism. While the latter property, together with the tropism for injured sites, can be exploited in the field of regenerative medicine, the homing of MSCs, engineered to carry anti-proliferative or pro-apoptotic signals, to cancer may be important for the development of anticancer therapy approaches.

The increasing interest around adult MSCs is further triggered by at least two additional characteristics: the immunoregulatory properties of these cells and their homing ability and specificity (Figure 1).

### Immunoregulatory properties of MSCs

One of the best-described functional properties of MSCs *in vivo* is their potent effect on the immune system. Indeed, it is well-known that MSCs have the capacity to suppress the immune response (Jiang et al., 2005; Corcione et al., 2006; Casiraghi et al., 2008; Jarvinen et al., 2008; Sheng et al., 2008). However, it has also been demonstrated that they can function as antigen presenting cells (Chan et al., 2006; Stagg et al., 2006).

The latter property of MSCs has been exploited successfully in a therapeutic setting to overcome graft versus host disease after haemopoietic-stem-cell transplantation (Le Blanc et al., 2008). In addition, MSCs have been used to limit inflammation in Crohn's diseases (Garcia-Olmo et al., 2005), and to reduce autoimmune side-effects following engraftment (Christopeit et al., 2008). Numerous characteristics contribute to the immunosuppressive effect of MSCs. Besides being characterized by low expression of Major Histocompatibility Complex class II (MHCII) and costimulatory molecules (B7-1 and B7-2), they interfere with various pathways of the immune response by means of cell-to-cell interactions and secretion of soluble factors, including members of the transforming growth factor-β family, interleukins 6 and 10, matrix metalloproteinases (MMPs), nitric oxide and indoleamine 2,3 deoxygenase (IDO). Different studies have reported the ability of MSCs to suppress T-cell proliferation, most likely via Prostaglandin E_2_ (PGE_2_) production (Jarvinen et al., 2008), to induce the T regulatory cells (Casiraghi et al., 2008), and to express co-inhibitory molecules as B7-H1 on their surface upon IFN-γ treatment (Sheng et al., 2008). Moreover, MSCs can impair maturation and function of dendritic cells and inhibit the proliferation, the differentiation and the chemotaxis of B-cells *in vitro* (Aggarwal and Pittenger, 2005; Beyth et al., 2005; Jiang et al., 2005; Corcione et al., 2006).

The immune-stimulating properties of these adult stem cells have been less investigated and seem to depend on the production of pro-inflammatory cytokines (Rasmusson et al., 2007). The dual immunoregulatory function of MSCs has been proposed to be cell dose-dependent, since high numbers of MSCs suppress whereas very low numbers seem to stimulate lymphocyte proliferation (Le Blanc et al., 2003). This latter observation has important implications in the use of MSCs as cell-therapeutics, as the cell dose is critical for the *in vivo* function and may rely on factors that are not well-understood, thereby limiting widespread use in the clinic.

### Homing of MSCs

An important distinguishing feature of MSCs compared to most other cell-types is that MSCs retain the ability to migrate to differentiated tissues. A number of telling studies have clearly demonstrated that when MSCs are systemically or locally administered, they selectively home to sites of injury and cancer (Ortiz et al., 2003; Rojas et al., 2005; Kidd et al., 2009). In pathological conditions an increase of circulating MSCs can be observed, suggesting the existence of a reservoir of mesenchymal cells that are mobilized in response to injury to target the damaged site and aid in tissue repair (Alm et al., 2010; Deng et al., 2011).

Why MSCs specifically home to these sites and what damaged and cancerous tissues have in common that attract MSCs are still open questions, but inflammation is most likely the responsible denominator. The high concentration of inflammatory chemokines released after tissue damage can indeed control the migration of MSCs, which express receptors for a number of grow factors including PDGF and IGF-1, and chemokines receptors, as CCR2, CCR3, CCR4, and CCL5 (Ponte et al., 2007). On the other hand, strong connections exist between tissue injury, chronic inflammation and cancer, as first described by Mina Bissell's group (Dolberg et al., 1985), so that tumors have been defined “wounds that do not heal” (Dvorak, 1986), where inflammatory cytokines and chemokines are produced and can drive MSC homing (Birnbaum et al., 2007; Dwyer et al., 2007; Menon et al., 2007).

The current knowledge about the mechanisms driving MSC migration and homing comes from studies on leukocytes (Butcher, 1991) and HSCs (Voermans et al., 1999). The initial adhesive interactions between circulating leukocytes and endothelial cells, called “rolling contacts,” are mediated by selectins (Lawrence and Springer, 1991). Next, the activation of integrin adhesiveness by chemokines determines the formation of more firm contacts that ultimately lead to extravasation (Lewinsohn et al., 1987). Bone marrow-derived MSCs express various integrins on their surface, among which integrin α4/β1, which mediates cell-cell and cell-extracellular matrix interactions by binding to vascular cell adhesion molecule (VCAM)-1 and to the V-region of fibronectin, respectively. In damaged tissues fibronectin is deposed together with fibrin at the injured sites to stop the bleeding. The provisional matrix is then remodeled by macrophages and fibroblasts, determining an increase in V region-exposing fibronectin, which, in turn, allows MSCs to adhere and transmigrate into the extracellular matrix (Valenick et al., 2005). Among the chemotactic chemokines involved in MSC homing, stromal cell-derived factor 1 (SDF-1) seems to play an important role. Although only low levels of the SDF-1 receptor, CXCR4, are present on the surface of MSCs, high intracellular levels of the receptor have been detected and seem to function as a reservoir. Indeed intracellular CXCR4 can be translocated to the membrane upon chemokine stimulation, thus contributing to the migration of MSCs (Wang et al., 2001; Wynn et al., 2004). Moreover, MSCs are able to secrete different metalloproteinases, including MMP-2 and MT1-MMP, which degrade the extracellular matrix barriers and allow extravasation and subendothelial migration (Ries et al., 2007).

The precise mechanisms driving MSC homing are still unclear, but represent a very attractive subject of investigation because of their implications in the therapeutic applications of these cells, as both reparative effectors and vectors of specific signals.

### MSCs in regenerative medicine

The unique characteristics of MSCs, such as their multipotency, immunological properties, homing and effects on tissue repair, raised expectations on the possibility to exploit these cells for therapeutic approaches. Indeed, MSCs are readily isolated from bone marrow and fat tissue (Lee et al., 2004), and can be administered to patients in an autologous manner, thus preventing rejection by the immune system.

MSCs have been extensively studied and already clinically tested for their role in bone repair and regeneration. Allogeneic MSCs have been used for the treatment of bone disorders as osteogenesis imperfecta (Horwitz et al., 2002; Le Blanc et al., 2005; Otsuru et al., 2012). For bone tissue engineering applications, these cells are used in combination with “scaffolds” that are designed to allow cell adhesion, survival and growth and that are even functionalized to provide cells with pro-osteogenic stimuli (Warnke et al., 2004; Marcacci et al., 2007). The advantage of using mesenchymal osteogenic precursors relies not only on the ability of these cells to differentiate into osteoblasts, but also on their capacity to provide trophic signals as growth factors and cytokines to the damaged tissues, thereby accelerating the regeneration process (Ciapetti et al., 2012).

Apart from bone-repair MSCs are also used to treat cardiovascular diseases. In particular acute myocardial infarction has been an important area of study to exploit MSC-based therapies. Cell death due to ischemia leads to decreased contractility of the heart. The general lack of an effective intrinsic mechanism to repair such damage prompted researchers to investigate both *in vitro* and *in vivo* the ability of MSCs to differentiate into cardiomyocytes (Toma et al., 2002; Wang et al., 2006). However, as mentioned before, there is currently no clear consensus if MSCs have the ability to differentiate into cardiomyocytes and, if so, by what signals. Experiments conducted by intravenously injecting MSCs in rodents showed that the majority of cells are “trapped” in the lungs (Schrepfer et al., 2007; Fischer et al., 2009). Moreover, only a small percentage of MSCs administered in swines using different delivery approaches is retained in the heart 2 weeks after transplantation (Freyman et al., 2006). For these reasons, it is believed that the positive effects of MSCs on damaged heart, may not be solely due to their ability to differentiate into cardiomyocytes. Instead, the release of trophic factors together with the suppression of inflammation may also be responsible for the healing effects of MSCs.

MSCs are also used for the treatment of neuronal injury and neurodegenerative diseases such as Alzheimer's, Parkinson's and Huntington's diseases. In this case, the reparative potential could depend on the ability of MSCs to locally secrete high amounts of brain-derived neurotrophic factor (BDNF), nerve growth factor (NGF), vascular endothelial growth factor (VEGF) and hepatocyte growth factor (HGF), indeed *in vitro* experiments have shown that the expression of these factors increases when MSCs are exposed to injured brain extracts (Chen et al., 2002). Moreover, the ability of MSCs to modulate the immune response might be crucial for neurodegenerative diseases characterized by chronic inflammation (Lee et al., 2010). However, different studies have also suggested the trans-differentiation of bone marrow-derived MSCs into neuronal-like cells under specific induction *in vitro* (Tondreau et al., 2008; Trzaska and Rameshwar, 2011).

Finally, MSCs are able to reverse acute kidney injury in mouse models. Also in this case the precise mechanisms by which MSCs protect from tissue damage is not understood. While initial studies demonstrated that trans-differentiation of the administered MSCs into tubular epithelium cells was responsible for the structural and functional repair of the kidney (Morigi et al., 2004), following experimental evidences revealed that only 2.0–2.5% of MSCs were actually engrafted (Herrera et al., 2007). Therefore, as suggested by additional studies in rodents, the release of factors that can regulate the immune response and have trophic, pro-angiogenic and mitogenic activities is the most accepted mechanism of action of MSCs in kidney repair (Tögel et al., 2005; Semedo et al., 2009).

### MSCs in anticancer therapy

While the potential of using MSCs in regerative medicine is releatively well-established, the use of MSCs in anticancer therapy is receiving increasing attention. Because MSCs have a clear capacity to home specifically to tumor sites in humans, they could be used as specialized delivery vehicles for targeted anticancer drugs or gene-therapy (Kidd et al., 2009, 2010; Loebinger et al., 2009; Sasportas et al., 2009; Yang et al., 2009). Nonetheless, this putative approach raises many (safety) questions because, although MSCs have intrinsic anti-tumorigenic activities, they also hold pro-tumorigenic properties, as suppressing the immune response and expressing growth factors and pro-angiogenic molecules that can aid in the formation of cancer stem cell niches (Roorda et al., 2009). Grisendi et al. already designed a novel cancer therapy strategy relying on the use of adipose-derived mesenchymal progenitors (AD-MSCs) as cellular vectors of a pro-apoptotic signal, i.e., tumor necrosis factor-related apoptosis-inducing ligand (TRAIL). When injected intravenously or subcutaneously into mice, TRAIL-transduced AD-MSCs were able to localize into tumors and mediate tumor cell apoptosis without apparent toxicities to normal tissues (Grisendi et al., 2010). Whether this strategy may also be suitable to eradicate human tumors awaits to be studied.

### Limitations of stem cell therapy

The use of stem cells for the therapy of human diseases raised several concerns in the past decade that proved a challenging objective to overcome. The result of the interaction between adult stem cells and target microenvironment needs to be further investigated before we can rule out potential risks for human health and obtain effective approaches for regenerative medicine.

Some of the challenges concerning transplanted MSCs are immune-mediated rejection, senescence-induced genetic instability or loss of function, and limited cell survival (Lim et al., 2011). Besides these issues, the major problem in using MSCs for clinical applications is the possibility of malignant transformation. The production of a sufficient amount of MSCs for clinical use requires a consistent *in vitro* expansion, which can lead to spontaneous transformation of the cells (Rubio et al., 2008). The exact mechanisms of MSC transformation are not completely understood, but c-myc upregulation, p-16 repression and increased telomerase activity seem to be involved. Furthermore, genetic manipulations of MSCs for the treatment of different diseases can *per se* increase the oncogenic potential of the cells, either because the transgene may be tumorigenic or because it might cause disruptions in the genome. MSCs have been found in a number of tumors including gastric adenocarcinoma (Xu et al., 2011), lipoma (Lin et al., 2007) and osteosarcoma (Brune et al., 2011), strongly suggesting their involvement in tumor development, and, importantly, various studies indicate these cells as potential sources of tumor associated fibroblasts (TAFs) (Kidd et al., 2012).

In the light of these observations, the choice of translating the potential of MSCs to the clinic should be cautiously considered.

## MSC released vesicles as a novel approach of cell-free therapy

In spite of the multipotent and self-renewal potential of MSCs and beyond the somewhat controversial ability of these cells to trans-differentiate into lineages of other germ layers, MCS have clear beneficial effects in the reparative processes of injured tissues. Experimental studies showed that only a small proportion of MSCs, locally or systemically administered, will actually be incorporated into injured tissues (Rosario et al., 1997; Li et al., 2008), indicating that the beneficial effects in tissue repair and regeneration is more likely indirect and depends on the paracrine activity of MSCs and not on their engraftment.

This intriguing hypothesis opens novel therapeutic perspectives aimed at the development of cell-free strategies based on the use of MSC secretome as a safe and potentially more advantageous alternative to cell-therapy approaches. While the soluble secretome of MSCs is partly characterized (Parekkadan et al., 2007; Lee et al., 2010; Roche et al., 2012), it seems unlikely that specific cytokines and growth factors alone give MSCs their remarkable healing abilities.

### Exosomes and microvesicles

Besides the long-time notion of growth factors and cytokines being an important part of the cellular secretome, it now appears that most, if not all cells, secrete large amounts of micro- and nano-vesicles, either constitutively or upon activation signals. The biochemical composition, the complex biogenesis of these vesicles and, in particular, their physiological role have only partially been unraveled. Yet, their potential as mediators of cell communication has not gone unnoticed, since these vesicles have remarkable features, including the ability to transfer proteins and functional genetic material such as RNA to other cells (Ratajczak et al., 2006; Valadi et al., 2007; Skog et al., 2008; Pegtel et al., 2010).

**Figure 2:**
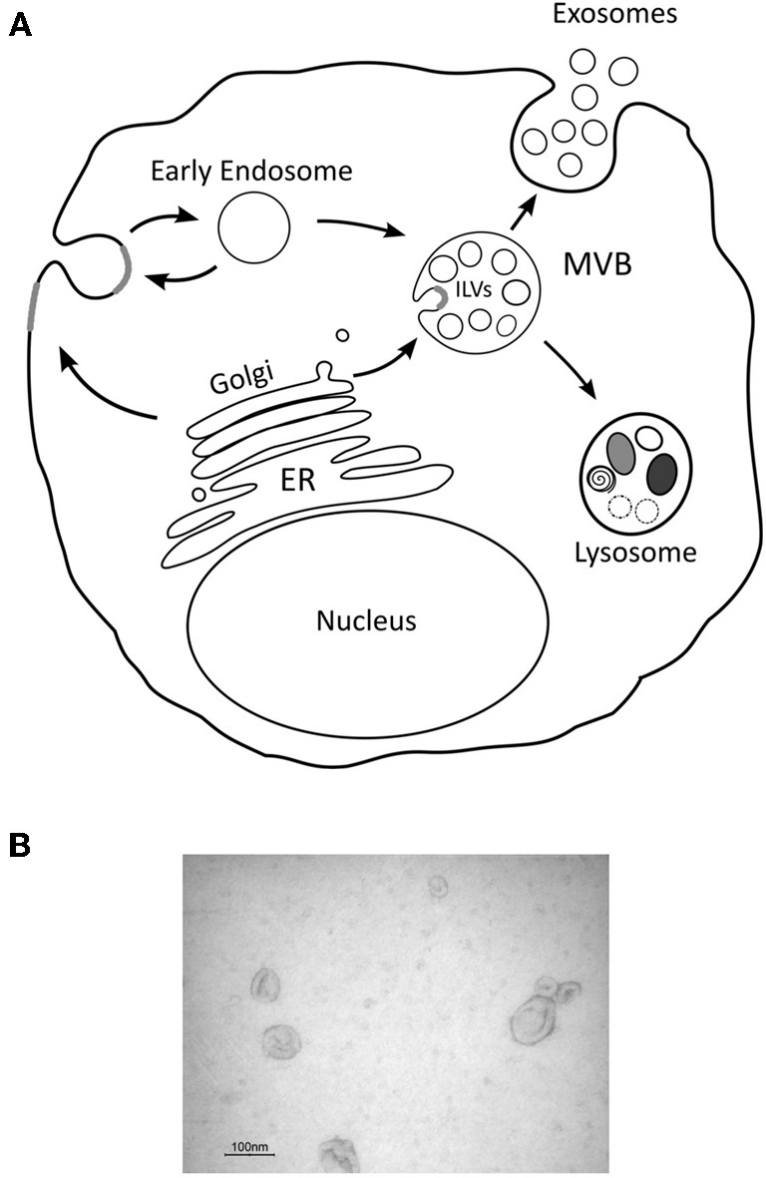
**Schematic representation of exosome biogenesis.** Intraluminal vesicles (ILVs) are generated by the inward budding of the limiting membrane of a subgroup of late endosomes called multivesicular bodies (MVBs). MVBs can be directed towards the cell periphery and, after fusion with the plasma membrane, release their content in the extracellular space. Secreted ILVs, now called “exosomes,” are then taken up by target cells **(A)**. Electron microscopy picture of exosomes isolated by differential ultracentrifugation **(B)**.

In particular exosomes have received much attention as these are a subclass of (nano)vesicles (50–100 nm) that are derived from specialized intracellular compartments known as late endosomes or Multi-vesicular bodies (MVBs). Many other types of vesicles exist that presumably derive from the plasma membrane and consensus has been reached to collectively name these extracellular membrane vesicles. Exosomes are released from most cells constitutively, but following activation their release is significantly increased. They were first implicated in reticulocyte maturation and later shown to have an important role in immune responses. More recently exosomes have been found in different biological fluids such as urine, plasma, malignant and pleural effusions of ascites and synovial fluid, and, because of their specific content, have been proposed as suitable biomarkers of different diseases (Skog et al., 2008; Nilsson et al., 2009). The biogenesis of exosomes involves the formation of intraluminal vesicles (ILV) by inward budding of the limiting membrane of MVBs. It is presumed, although many molecular details are lacking, that MVBs fuse with the plasma membrane to release the ILVs as exosomes (Figure 2). Once secreted exosomes can either be taken up by target cells localized in proximity of the cell of origin or travel to more distant sites through the blood and possibly other biological fluids. Théry et al. (2006) provided a detailed description of the most recognized procedures to isolate and characterize exosomes from cell supernatant and bodily fluids. The development and use of standardized protocols is critical because other kinds of vesicles as well as membrane fragments are normally present in the starting material and can contaminate exosome preparations. Mechanistically, exosomes, but also other types of microvesicles, can operate in a multitude of ways since they can be considered as complex vectors that can hold essentially all known biological molecules and likely the solutes that are present in the parental cells. These molecules include, but are not restricted to, proteins (both ubiquitous and cell-specific), mRNAs, microRNAs (miRNAs) and lipid molecules.

Given the multiplicity of signals carried by these vesicles through the horizontal transfer of functional RNAs and proteins, their implication in various diseases and especially in cancer is being intensively investigated. It is becoming more and more evident that cancer cells exploit exosome-mediated signaling to modify their microenvironment, but also to exert systemic functions. Indeed exosomes can promote the formation of pre-metastatic niches, thereby optimising the conditions for tumor spreading (Hood et al., 2011). Moreover, the amount and the content of exosomes consistently vary based on the microenvironmental conditions, and particularly when cells are subjected to stress factors (Parolini et al., 2009; Hedlund et al., 2011; Lv et al., 2012). For instance, the acidic extracellular pH associated with the aggressiveness and chemoresistance of various solid tumors (Simon et al., 1993; Mahoney et al., 2003; Nishisho et al., 2011) is able to increase exosome release and uptake (Parolini et al., 2009).

**Figure 3:**
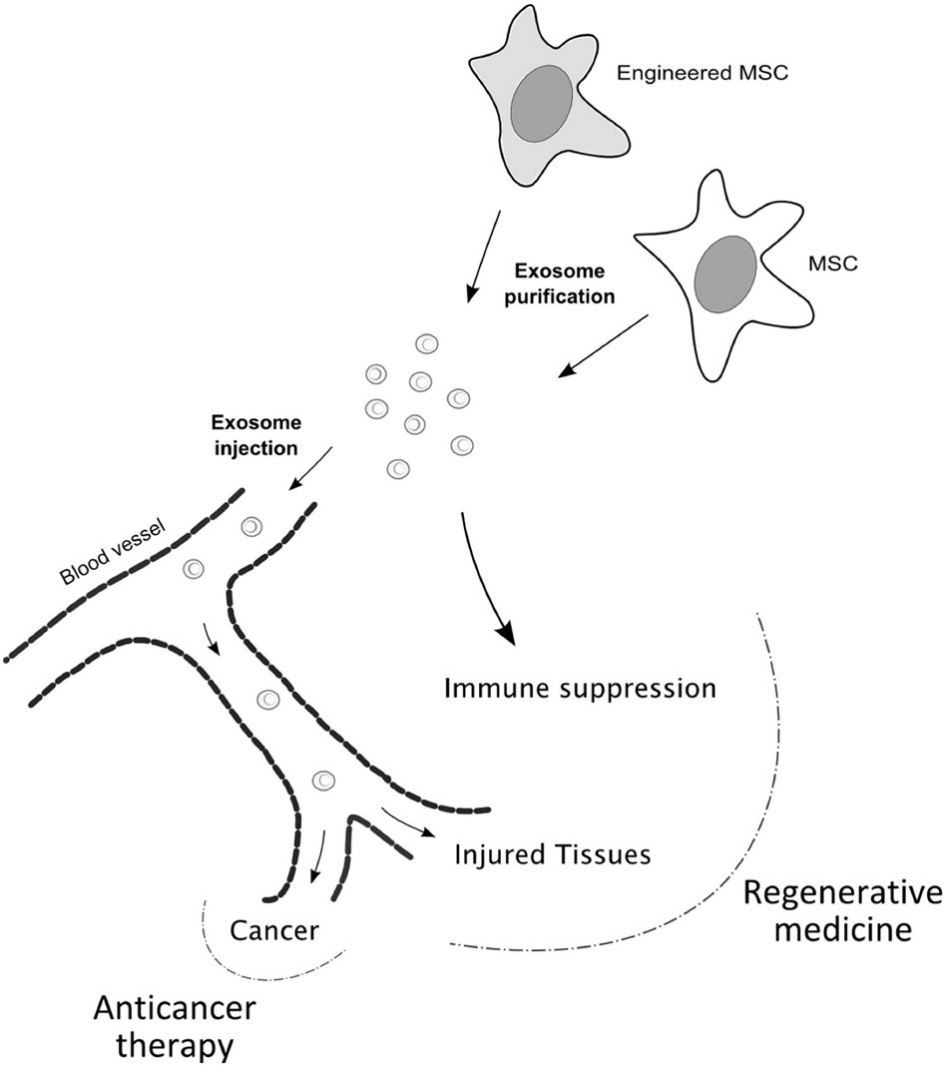
**Proposed model for therapeutic applications of MSC-derived exosomes.** MSC-derived exosomes may be used instead of MSCs in regenerative medicine and anticancer therapy, since they seem to hold the beneficial properties of the parental cells. MSC-derived exosomes might retain the homing ability displayed by MSCs towards sites of inflammation and function in tissue repair, also by modulating the immune response. Concerning anticancer therapy, exosomes derived from engineered MSCs might be used to mediate anti-proliferative or pro-apoptotic effects. Moreover, whenever the tropism of exosomes would not be retained, MSCs may be modified to confer specific targeting to exosomes.

On the other hand, the sophisticated make up of exosomes, which strongly suggests an important role in cell-cell communication, opens novel perspectives in exploiting these vesicles in therapeutic settings. Exosomes might be isolated from cells that hold promising therapeutic applications, as MSCs in regenerative medicine, and systemically or locally administered to mimic the effect of the parental cell. Whether MSC-derived exosomes retain the homing properties of the cells of origin is still largely unknown and is an important question to be answered, although *in vivo* studies have shown beneficial effects of intravenously injected exosomes in tissue repair. Moreover, exosomes can be used as targeted delivery vehicles of therapeutic miRNAs. Alvarez-Erviti et al. (2011) succeeded in delivering functional siRNA to the mouse brain by systemically injecting targeted exosomes. To confer tissue-specificity to exosomes the authors engineered low immunogenic cells to express an exosomal membrane protein, Lamp2b, fused to the neuron-specific RVG peptide. Exosomes were then isolated and loaded with exogenous siRNAs by electroporation. Considering the reparative, immune suppressive and homing properties of MSCs, the use of exosomes derived from these cells modified to express high levels of specific miRNAs could also be considered, once ascertained that the miRNAs of interest are actually enriched in the exosomal compartment. In case the tropism of exosomes would not reflect that of MSCs or if different targeting would be required, exosomes bearing tissue-specific receptor on their surface could be engineered (Alvarez-Erviti et al., 2011), or local administration might be considered (Figure 3).

### Characterization of MSC-released vesicles

Despite the interest raised by MSC-derived microvesicles for their potential role in physiological and pathological conditions, and for their possible applications in the treatment of various diseases, only few studies have been conducted on the (specific) RNA and protein content of these vesicles.

The presence of selected miRNAs within MSC-derived microvesicles has been proposed by Collino et al. (2010). In these studies a comparative miRNA profiling was performed with arrays using bone marrow and tissue specific (liver) MSCs and their respective microvesicles. The authors found that some miRNAs were present both in microvesicles and in the cells of origin. However, some miRNAs appeared to have been selectively sorted into the MVs, as these were not detectable in the cells, while, on the contrary, others were present in the cells but not in the MVs. These observations support the existence of a mechanism that controls the sorting of miRNAs in MSC vesicles. Nevertheless, without accurate quantitation by either deep-sequencing techniques and/or quantitative RT-PCR in combination with functional experiments, the biological relevance of these findings remain unclear. The only way to explain that miRNAs are present in exosomes and not in the producing cells is that the mature RNA species are rapidly secreted, having no chance to accumulate within the cytoplasm with the risk of being degraded. Specific miRNAs might be produced by these cells only for the purpose of cell–cell communication, without ever reaching a critical level in the cell of origin to exert a function, being repressing the translation of particular target mRNAs. Although this is possible in theory, no experimental data is available. In contrast, the sorting of specific signaling molecules (proteins) into exosomes does seem to have a clear effect on the producing cells (Chairoungdua et al., 2010; Verweij et al., 2011) and may also be of importance *in vivo* (Al-Nedawi et al., 2009; Peinado et al., 2012). However, it does appear that microRNAs may be specifically transported into cells for specialized functions while the target cells seems to lack these microRNAs (Rader and Parmacek, 2012).

Based on the Gene Ontology analysis, the predicted and validated targets of the miRNAs detected in both MSCs and MSC-MVs are related to development, cell survival and differentiation, while some MSC MV-enriched miRNAs were more associated with the regulation of the immune system. Moreover, microvesicle-derived miRNAs transferred to recipient cells were able to suppress specific targets, thus confirming the functionality of these mediators in cell-to-cell communication (Collino et al., 2010).

Besides the genetic content of MVs, the proteome may be equally important. Only one study has been conducted so far that characterized the protein composition of MSC-derived MVs in more detail. By characterizing the content of bone marrow MSC-derived MVs, Kim et al. identified 730 proteins, among which mediators controlling self-renewal and differentiation. Interestingly, their analysis revealed a number of surface markers such as PDGFRB, EGFR, and PLAUR, signaling molecules of RAS-MAPK, RHO, and CDC42 pathways, cell adhesion molecules and additional MSC antigens that support a possible role for such vesicles in tissue repair (Kim et al., 2012). Based on these results, it appears that MSC-MVs hold many of the characteristics of the MSCs themselves, and may be important for the function of these adult stem cells *in vivo* besides the classic secreted factors.

### MSC-released vesicles in regenerative medicine and cancer

While the predominant role of MSC paracrine activity in tissue repair has already been established, whether MSC-MVs also have a role remains to be studied.

The protective paracrine activity of MSCs in kidney injury fostered several studies into the potential contribution of MSC-derived microvesicles in renal repair. Microvesicles released by MSCs protect against renal injury in the mouse remnant kidney model, support renal repair in ischemia/reperfusion-induced acute kidney injury (AKI), and protect from lethal cisplatin-induced AKI, most likely by inhibiting apoptosis of tubular epithelial cells (Gatti et al., 2011; Bruno et al., 2012; He et al., 2012).

In animal models of intervertebral disc (IVD) degeneration MSCs have been demonstrated able to restore the normal disc structure. Since IVD degeneration seems to depend on alterations of nucleus pulposus (NP) cells, Strassburg et al. (2012) investigated the interactions between MSCs and degenerate NP cells and found that the two cell types primarily communicate via an extensive direct transfer of membrane components and via microvesicles.

The potential use of MSC-MVs for the treatment of cardiovascular diseases has recently been reviewed by Lai et al. (2011). The authors previously demonstrated the therapeutic activity of MVs isolated from embryonic stem cell-derived MSCs (ESC-MSCs) in a mouse model of myocardial ischemia/reperfusion (Lai et al., 2010). They suggest that the secretion of protective exosomes is a general property and perhaps a predominant function of MSCs, probably related to the supporting role of the stromal cells. Considering the limitations and costs related to the use of embryonic stem cells and the high amount of cells required for MV production, the same group also generated MYC-immortalized ESC-MSCs and demonstrated that MVs derived from these cells still display their original cardioprotective activity (Chen et al., 2011).

If the beneficial and protective effects of MSC-MVs in tissue repair have been reported in different pathological conditions, their use for cancer therapy needs careful consideration.

Bone marrow MSC-derived exosomes have been shown to support tumor growth and angiogenesis in a mouse xenograft model of gastric carcinoma, and the pro-angiogenic effect has been ascribed to the increase of VEGF expression in tumor cells (Zhu et al., 2012). This evidence is not completely unexpected since MSCs have been reported to have various tumor promoting functions (Roorda et al., 2009), and highlights once more that it is mandatory to be cautious when evaluating the risks related to the use of engineered MSCs or MSC-derived exosomes in anticancer therapy. Finally, Kyo Won Lee's group demonstrated that both breast and ovarian cancer cells (Cho et al., 2011, 2012) can condition AD-MSCs to generate tumor associated myofibrobasts. It might be interesting to investigate whether, in turn, MSC-derived myofibroblast-like cells, or pre-conditioned MSCs exploit the same mechanism to further support tumor development.

## Concluding remarks

While the use of MSCs in regenerative medicine and anti-cancer treatment raised high expectations, concerns about safety and tight regulations hampered their practical use in clinical settings. However, the use of MSC-derived secretome and, in particular, of the vesicles released by these cells may have many advantages compared to a cell-based approach.

The increasing interest around this strategy of intercellular crosstalk adopted by MSCs relies on the ability of these vesicles to condition and reprogram the surrounding microenvironment, thereby influencing a variety of biological responses, in particular in injured tissues and cancer.

MVs function primarily in cell–cell communication, which is, as discussed above, highly relevant in the biology of MSCs. The significant contribution of MSC paracrine activity, rather than their ability to differentiate, to the reparative process has already been established. It will now be of importance to decipher the exact role of MSC secretome and vesicles, since it is likely that in parallel to soluble factors as growth factors and cytokines, MVs strongly contribute to the paracrine effects of these cells. Indeed MVs present a complex composition that mirrors that of the parental cells and seems to have similar properties *in vivo*.

Therefore, the use of MSC secretome-derived vesicles represents an interesting alternative for tissue repair that might overcome the limitations and risks associated to cell-therapy approaches. Concerning the potential applications for cancer therapy, exosomes released by opportunely engineered MSCs may still retain the ability to home to tumor site and, at the same time, mediate anti-proliferative or pro-apoptotic effects, relieving the concerns related to the genetic manipulation of stem cells for cell-therapy.

Evidently, crucial questions need to be answered before the objective described above can be satisfactorily fulfilled. First, it is necessary to verify to which extent MSC micro- and nano-vesicles contribute to the beneficial effects mediated by MSCs; second, the content of these vesicles, in terms of proteins and, in particular, small RNAs, needs to be thoroughly characterized; and, finally, it is essential to uncover the effect of the genetic content of MSC-MVs on recipients cells and determine which cellular pathways may be affected.

### Conflict of interest statement

The authors declare that the research was conducted in the absence of any commercial or financial relationships that could be construed as a potential conflict of interest.

## References

[B1] Aggarwal S. Pittenger M. F. (2005). Human mesenchymal stem cells modulate allogeneic immune cell responses. Blood 105, 1815–1822 10.1182/blood-2004-04-1559 15494428

[B2] Al-Nedawi K. Meehan B. Kerbel R. S. Allison A. C. Rak J. (2009). Endothelial expression of autocrine VEGF upon the uptake of tumor-derived microvesicles containing oncogenic EGFR. Proc. Natl. Acad. Sci. U.S.A. 106, 3794–3799 10.1073/pnas.0804543106 19234131 PMC2656159

[B3] Alm J. J. Koivu H. M. Heino T. J. Hentunen T. A. Laitinen S. Aro H. T. (2010). Circulating plastic adherent mesenchymal stem cells in aged hip fracture patients. J. Orthop. Res. 28, 1634–1642 10.1002/jor.21167 20540091

[B4] Alvarez-Erviti L. Seow Y. Yin H. Betts C. Lakhal S. Wood M. J. (2011). Delivery of siRNA to the mouse brain by systemic injection of targeted exosomes. Nat. Biotechnol. 29, 341–345 10.1038/nbt.1807 21423189

[B5] Beyth S. Borovsky Z. Mevorach D. Liebergall M. Gazit Z. Aslan H. Galun E. Rachmilewitz J. (2005). Human mesenchymal stem cells alter antigen-presenting cell maturation and induce T-cell unresponsiveness. Blood 105, 2214–2219 10.1182/blood-2004-07-2921 15514012

[B6] Birnbaum T. Roider J. Schankin C. J. Padovan C. S. Schichor C. Goldbrunner R. Straube A. (2007). Malignant gliomas actively recruit bone marrow stromal cells by secreting angiogenic cytokines. J. Neurooncol. 83, 241–247 10.1007/s11060-007-9332-4 17570034

[B7] Bosch P. Musgrave D. S. Lee J. Y. Cummins J. Shuler T. Ghivizzani T. C. Evans T. Robbins T. D. Huard. (2000). Osteoprogenitor cells within skeletal muscle. J. Orthop. Res. 8, 933–944 10.1002/jor.1100180613 11192254

[B8] Brune J. C. Tormin A. Johansson M. C. Rissler P. Brosjö O. Löfvenberg R. von Steyern F. V. Mertens F. Rydholm A. Scheding S. (2011). Mesenchymal stromal cells from primary osteosarcoma are non-malignant and strikingly similar to their bone marrow counterparts. Int. J. Cancer 129, 319–330 10.1002/ijc.25697 20878957

[B9] Bruno S. Grange C. Collino F. Deregibus M. C. Cantaluppi V. Biancone L. Tetta C. Camussi G. (2012). Microvesicles derived from mesenchymal stem cells enhance survival in a lethal model of acute kidney injury. PLoS ONE 7:e33115 10.1371/journal.pone.0033115 22431999 PMC3303802

[B10] Butcher E. C. (1991). Leukocyte-endothelial cell recognition: three (or more) steps to specificity and diversity. Cell 67, 1033–1036 10.1016/0092-8674(91)90279-8 1760836

[B11] Caplan A. I. (1991). Mesenchymal stem cells. J. Orthop. Res. 9, 641–650 10.1002/jor.1100090504 1870029

[B12] Casiraghi F. Azzollini N. Cassis P. Imberti B. Morigi M. Cugini D. Cavinato R. A. Todeschini M. Solini S. Sonzogni A. Perico N. Remuzzi G. Noris M. (2008). Pretransplant infusion of mesenchymal stem cells prolongs the survival of a semiallogeneic heart transplant through the generation of regulatory T cells. J. Immunol. 181, 3933–3946 18768848 10.4049/jimmunol.181.6.3933

[B13] Chairoungdua A. Smith D. L. Pochard P. Hull M. Caplan M. J. (2010). Exosome release of β-catenin: a novel mechanism that antagonizes Wnt signaling. J. Cell Biol. 190, 1079–1091 10.1083/jcb.201002049 20837771 PMC3101591

[B14] Chan J. L. Tang K. C. Patel A. P. Bonilla L. M. Pierobon N. Ponzio N. M. Rameshwar P. (2006). Antigen-presenting property of mesenchymal stem cells occurs during a narrow window at low levels of interferon-{gamma}. Blood 107, 4817–4824 10.1182/blood-2006-01-0057 16493000 PMC1895812

[B15] Chen T. S. Arslan F. Yin Y. Tan S. S. Lai R. C. Choo A. B. Padmanabhan J. Lee C. N. de Kleijn D. P. Lim S. K. (2011). Enabling a robust scalable manufacturing process for therapeutic exosomes through oncogenic immortalization of human ESC-derived MSCs. J. Transl. Med. 9, 47 10.1186/1479-5876-9-47 21513579 PMC3100248

[B16] Chen X. Li Y. Wang L. Katakowski M. Zhang L. Chen J. Xu Y. Gautam S. C. Chopp M. (2002). Ischemic rat brain extracts induce human marrow stromal cell growth factor production. Neuropathology 22, 275–279 12564767 10.1046/j.1440-1789.2002.00450.x

[B17] Cho J. A. Park H. Lim E. H. Kim K. H. Choi J. S. Lee J. H. Shin J. W. Lee K. W. (2011). Exosomes from ovarian cancer cells induce adipose tissue-derived mesenchymal stem cells to acquire the physical and functional characteristics of tumor-supporting myofibroblasts. Gynecol. Oncol. 123, 379–386 10.1016/j.ygyno.2011.08.005 21903249

[B18] Cho J. A. Park H. Lim E. H. Lee K. W. (2012). Exosomes from breast cancer cells can convert adipose tissue-derived mesenchymal stem cells into myofibroblast-like cells. Int. J. Oncol. 40, 130–138 10.3892/ijo.2011.1193 21904773

[B19] Christopeit M. Schendel M. Foll J. Muller L. P. Keysser G. Behre G. (2008). Marked improvement of severe progressive systemic sclerosis after transplantation of mesenchymal stem cells from an allogeneic haploidentical-related donor mediated by ligation of CD137L. Leukemia 22, 1062–1064 10.1038/sj.leu.2404996 17972956

[B20] Ciapetti G. Granchi D. Baldini N. (2012). The combined use of mesenchymal stromal cells and scaffolds for bone repair. Curr. Pharm. Des. 18, 1796–1820 22352754 10.2174/138161212799859648

[B21] Collino F. Deregibus M. C. Bruno S. Sterpone L. Aghemo G. Viltono L. Tetta C. Camussi G. (2010). Microvesicles derived from adult human bone marrow and tissue specific mesenchymal stem cells shuttle selected pattern of miRNAs. PLoS ONE 5:e11803 10.1371/journal.pone.0011803 20668554 PMC2910725

[B22] Corcione A. Benvenuto F. Ferretti E. Giunti D. Cappiello V. Cazzanti F. Risso M. Gualandi F. Mancardi G. L. Pistoia V. Uccelli A. (2006). Human mesenchymal stem cells modulate B-cell functions. Blood 107, 367–372 10.1182/blood-2005-07-2657 16141348

[B23] Deng J. Zou Z. M. Zhou T. L. Su Y. P. Ai G. P. Wang J. P. Xu H. Dong S. W. (2011). Bone marrow mesenchymal stem cells can be mobilized into peripheral blood by G-CSF *in vivo* and integrate into traumatically injured cerebral tissue. Neurol. Sci. 32, 641–651 10.1007/s10072-011-0608-2 21678074

[B24] Dezawa M. Ishikawa H. Itokazu Y. Yoshihara T. Hoshino M. Takeda S. Ide C. Nabeshima Y. (2005). Bone marrow stromal cells generate muscle cells and repair muscle degeneration. Science 309, 314–317 10.1126/science.1110364 16002622

[B25] Dezawa M. Kanno H. Hoshino M. Cho H. Matsumoto N. Itokazu Y. Tajima N. Yamada H. Sawada H. Ishikawa H. Mimura T. Kitada M. Suzuki Y. Ide C. (2004). Specific induction of neuronal cells from bone marrow stromal cells and application for autologous transplantation. J. Clin. Invest. 113, 1701–1710 10.1172/JCI20935 15199405 PMC420509

[B26] Dolberg D. S. Hollingsworth R. Hertle M. Bissell M. J. (1985). Wounding and its role in RSV-mediated tumor formation. Science 230, 676–678 10.1126/science.2996144 2996144

[B27] Dominici M. Le Blanc K. Mueller I. Slaper-Cortenbach I. Marini F. Krause D. Deans R. Keating A. Prockop D. J. Horwitz E. (2006). Minimal criteria for defining multipotent mesenchymal stromal cells. The International Society for Cellular Therapy position statement. Cytotherapy 8, 315–317 10.1080/14653240600855905 16923606

[B28] Dvorak H. F. (1986). Tumors: wounds that do not heal. Similarities between tumor stroma generation and wound healing. N. Engl. J. Med. 315, 1650–1659 10.1056/NEJM198612253152606 3537791

[B29] Dwyer R. M. Potter-Beirne S. M. Harrington K. A. Lowery A. J. Hennessy E. Murphy J. M. Barry F. P. O'Brien T. Kerin M. J. (2007). Monocyte chemotactic protein-1 secreted by primary breast tumors stimulates migration of mesenchymal stem cells. Clin. Cancer Res. 13, 5020–5027 10.1158/1078-0432.CCR-07-0731 17785552

[B30] Fischer U. M. Harting M. T. Jimenez F. Monzon-Posadas W. O. Xue H. Savitz S. I. Laine G. A. Cox C. S. Jr. (2009). Pulmonary passage is a major obstacle for intravenous stem cell delivery: the pulmonary first-pass effect. Stem Cells Dev. 18, 683–692 10.1089/scd.2008.0253 19099374 PMC3190292

[B31] Freyman T. Polin G. Osman H. Crary J. Lu M. Cheng L. Palasis M. Wilensky R. L. (2006). A quantitative, randomized study evaluating three methods of mesenchymal stem cell delivery following myocardial infarction. Eur. Heart J. 27, 1114–1122 10.1093/eurheartj/ehi818 16510464

[B32] Friedenstein A. J. Chailakhjan R. K. Lalykina K. S. (1970). The development of fibroblast colonies in monolayer cultures of guinea-pig bone marrow and spleen cells. Cell Tissue Kinet. 3, 393–403 5523063 10.1111/j.1365-2184.1970.tb00347.x

[B33] Garcia-Olmo D. Garcia-Arranz M. Herreros D. Pascual I. Peiro C. Rodríguez-Montes J. A. (2005). A phase I clinical trials of the treatment of Crohn's fistula by adipose mesenchymal stem cell transplantation. Dis. Colon Rectum 48, 1416–1423 10.1007/s10350-005-0052-6 15933795

[B34] Gatti S. Bruno S. Deregibus M. C. Sordi A. Cantaluppi V. Tetta C. Camussi G. (2011). Microvesicles derived from human adult mesenchymal stem cells protect against ischaemia-reperfusion-induced acute and chronic kidney injury. Nephrol. Dial. Transplant. 26, 1474–1483 10.1093/ndt/gfr015 21324974

[B35] Grisendi G. Bussolari R. Cafarelli L. Petak I. Rasini V. Veronesi E. De Santis G. Spano C. Tagliazzucchi M. Barti-Juhasz H. Scarabelli L. Bambi F. Frassoldati A. Rossi G. Casali C. Morandi U. Horwitz E. M. Paolucci P. Conte P. Dominici M. (2010). Adipose-derived mesenchymal stem cells as stable source of tumor necrosis factor-related apoptosis-inducing ligand delivery for cancer therapy. Cancer Res. 70, 3718–3729 10.1158/0008-5472.CAN-09-1865 20388793

[B36] He J. Wang Y. Sun S. Yu M. Wang C. Pei X. Zhu B. Wu J. Zhao W. (2012). Bone Marrow stem cells-derived micro-vesicles protect against renal injury in the mouse remnant kidney model. Nephrology (Carlton) 17, 493–500 10.1111/j.1440-1797.2012.01589.x 22369283

[B37] Hedlund M. Nagaeva O. Kargl D. Baranov V. Mincheva-Nilsson L. (2011). Thermal- and oxidative stress causes enhanced release of NKG2D ligand-bearing immunosuppressive exosomes in leukemia/lymphoma T and B cells. PLoS ONE 6:e16899 10.1371/journal.pone.0016899 21364924 PMC3045385

[B38] Herrera M. B. Bussolati B. Bruno S. Morando L. Mauriello-Romanazzi G. Sanavio F. Stamenkovic I. Biancone L. Camussi G. (2007). Exogenous mesenchymal stem cells localize to the kidney by means of CD44 following acute tubular injury. Kidney Int. 72, 430–441 10.1038/sj.ki.5002334 17507906

[B39] Hood J. L. San R. S. Wickline S. A. (2011). Exosomes released by melanoma cells prepare sentinel lymph nodes for tumor metastasis. Cancer Res. 71, 3792–3801 10.1158/0008-5472.CAN-10-4455 21478294

[B40] Horwitz E. M. Gordon P. L. Koo W. K. Marx J. C. Neel M. D. McNall R. Y. Muul L. Hofmann T. (2002). Isolated allogeneic bone marrow-derived mesenchymal cells engraft and stimulate growth in children with osteogenesis imperfecta: implications for cell therapy of bone. Proc. Natl. Acad. Sci. U.S.A. 99, 8932–8937 10.1073/pnas.132252399 12084934 PMC124401

[B41] Jarvinen L. Badri L. Wettlaufer S. Ohtsuka T. Standiford T. J. Toews G. B. Pinsky D. J. Peters-Golden M. Lama V. N. (2008). Lung resident mesenchymal stem cells isolated from human lung allografts inhibit T cell proliferation via a soluble mediator. J. Immunol. 181, 4389–4396 18768898 10.4049/jimmunol.181.6.4389 PMC3644960

[B42] Jiang X. X. Zhang Y. Liu B. Zhang S. X. Wu Y. Yu X. D. Mao N. (2005). Human mesenchymal stem cells inhibit differentiation and function of monocyte-derived dendritic cells. Blood 105, 4120–4126 10.1182/blood-2004-02-0586 15692068

[B43] Johnson A. Dorshkind K. (1986). Stromal cells in myeloid and lymphoid long-term bone marrow cultures can support multiple hemopoietic lineages and modulate their production of hemopoietic growth factors. Blood 68, 1348–1354 3490887

[B44] Kidd S. Caldwell L. Dietrich M. Samudio I. Spaeth E. L. Watson K. Shi Y. Abbruzzese J. Konopleva M. Andreeff M. Marini F. C. (2010). Mesenchymal stromal cells alone or expressing interferon-beta suppress pancreatic tumors *in vivo*, an effect countered by anti-inflammatory treatment. Cytotherapy 12, 615–625 10.3109/14653241003631815 20230221

[B45] Kidd S. Spaeth E. Dembinski J. L. Dietrich M. Watson K. Klopp A. Battula V. L. Weil M. Andreeff M. Marini F. C. (2009). Direct evidence of mesenchymal stem cell tropism for tumor and wounding microenvironments using *in vivo* bioluminescent imaging. Stem Cells 27, 2614–2623 10.1002/stem.187 19650040 PMC4160730

[B46] Kidd S. Spaeth E. Watson K. Burks J. Lu H. Klopp A. Andreeff M. Marini F. C. (2012). Origins of the tumor microenvironment: quantitative assessment of adipose-derived and bone marrow-derived stroma. PLoS ONE 7:e30563 10.1371/journal.pone.0030563 22363446 PMC3282707

[B47] Kim H. S. Choi D. Y. Yun S. J. Choi S. M. Kang J. W. Jung J. W. Hwang D. Kim K. P. Kim D. W. (2012). Proteomic analysis of microvesicles derived from human mesenchymal stem cells. J. Proteome Res. 11, 839–849 10.1021/pr200682z 22148876

[B48] Lai R. C. Arslan F. Lee M. M. Sze N. S. Choo A. Chen T. S. Salto-Tellez M. Timmers L. Lee C. N. El Oakley R. M. Pasterkamp G. de Kleijn D. P. Lim S. K. (2010). Exosome secreted by MSC reduces myocardial ischemia/reperfusion injury. Stem Cell Res. 4, 214–222 10.1016/j.scr.2009.12.003 20138817

[B49] Lai R. C. Chen T. S. Lim S. K. (2011). Mesenchymal stem cell exosome: a novel stem cell-based therapy for cardiovascular disease. Regen. Med. 6, 481–492 10.2217/rme.11.35 21749206

[B50] Lawrence M. B. Springer T. A. (1991). Leukocytes roll on a selectin at physiologic flow rates: distinction from and prerequisite for adhesion through integrins. Cell 65, 859–873 10.1016/0092-8674(91)90393-D 1710173

[B51] Lewinsohn D. M. Bargatze R. F. Butcher E. C. (1987). Leukocyte-endothelial cell recognition: evidence of a common molecular mechanism shared by neutrophils, lymphocytes, and other leukocytes. J. Immunol. 138, 4313–4321 3584977

[B52a] Le Blanc K. Frassoni F. Ball L. Locatelli F. Roelofs H. Lewis I. Lanino E. Sundberg B. Bernardo M. E. Remberger M. Dini G. Egeler R. M. Bacigalupo A. Fibbe W. Ringdén O. (2008). Mesenchymal stem cells for treatment of steroid-resistant, severe, acute graft-versus-host disease: a phase II study. Lancet 371, 1579–1586 10.1016/S0140-6736(08)60690-X 18468541

[B52] Le Blanc K. Götherström C. Ringdén O. Hassan M. McMahon R. Horwitz E. Anneren G. Axelsson O. Nunn J. Ewald U. Nordén-Lindeberg S. Jansson M. Dalton A. Aström E. Westgren M. (2005). Fetal mesenchymal stem cell engraftment in bone after *in utero* transplantation in a patient with severe osteogenesis imperfecta. Transplantation 79, 1607–1614 15940052 10.1097/01.tp.0000159029.48678.93

[B53] Le Blanc K. Tammik L. Sundberg B. Haynesworth S. E. Ringdén O. (2003). Mesenchymal stem cells inhibit and stimulate mixed lymphocyte cultures and mitogenic responses independently of the major histocompatibility complex. Scand. J. Immunol. 57, 11–20 10.1046/j.1365-3083.2003.01176.x 12542793

[B54] Lee J. K. Jin H. K. Endo S. Schuchman E. H. Carter J. E. Bae J. S. (2010). Intracerebral transplantation of bone marrow-derived mesenchymal stem cells reduces amyloid-beta deposition and rescues memory deficits in Alzheimer's disease mice by modulation of immune responses. Stem Cells 28, 329–343 10.1002/stem.277 20014009

[B55] Lee M. J. Kim J. Kim M. Y. Bae Y. S. Ryu S. H. Lee T. G. Kim J. H. (2010). Proteomic analysis of tumor necrosis factor-alpha-induced secretome of human adipose tissue-derived mesenchymal stem cells. J. Proteome Res. 9, 1754–1762 10.1021/pr900898n 20184379

[B56] Lee R. H. Kim B. Choi I. Kim H. Choi H. S. Suh K. Bae Y. C. Jung J. S. (2004). Characterization and expression analysis of mesenchymal stem cells from human bone marrow and adipose tissue. Cell. Physiol. Biochem. 14, 311–324 10.1159/000080341 15319535

[B57] Leonardi E. Ciapetti G. Baglìo S. R. Devescovi V. Baldini N. Granchi D. (2009). Osteogenic properties of late adherent subpopulations of human bone marrow stromal cells. Histochem. Cell Biol. 132, 547–557 10.1007/s00418-009-0633-x 19711092

[B58] Li T. S. Takahashi M. Ohshima M. Qin S. L. Kubo M. Muramatsu K. Hamano K. (2008). Myocardial repair achieved by the intramyocardial implantation of adult cardiomyocytes in combination with bone marrow cells. Cell Transplant. 17, 695–703 10.3727/096368908786092702 18819257

[B59] Lim P. Patel S. A. Rameshwar P. (2011). Effective tissue repair and immunomodulation by mesenchymal stem cells within a milieu of cytokines, in Stem Cell-Based Tissue Repair, eds Gorodetsky R. Schaĺfer R. (Cambridge: RSC Publications), 346–365

[B60] Lin T. M. Chang H. W. Wang K. H. Kao A. P. Chang C. C. Wen C. H. Lai C. S. Lin S. D. (2007). Isolation and identification of mesenchymal stem cells from human lipoma tissue. Biochem. Biophys. Res. Commun. 361, 883–889 10.1016/j.bbrc.2007.07.116 17679141

[B61] Loebinger M. R. Kyrtatos P. G. Turmaine M. Price A. N. Pankhurst Q. Lythgoe M. F. Janes S. M. (2009). Magnetic resonance imaging of mesenchymal stem cells homing to pulmonary metastases using biocompatible magnetic nanoparticles. Cancer Res. 69, 8862–8867 10.1158/0008-5472.CAN-09-1912 19920196 PMC2833408

[B62] Lv L. H. Wan Y. L. Lin Y. Zhang W. Yang M. Li G. L. Lin H. M. Shang C. Z. Chen Y. J. Min J. (2012). Anticancer drugs cause release of exosomes with heat shock proteins from human hepatocellular carcinoma cells that elicit effective natural killer cell antitumor responses *in vitro*. J. Biol. Chem. 287, 15874–15885 10.1074/jbc.M112.340588 22396543 PMC3346092

[B63] Mahoney B. P. Raghunand N. Baggett B. Gillies R. J. (2003). Tumor acidity, ion trapping and chemotherapeutics. I. Acid pH affects the distribution of chemotherapeutic agents *in vitro*. Biochem. Pharmacol. 66, 1207–1218 10.1016/S0006-2952(03)00467-2 14505800

[B64] Marcacci M. Kon E. Moukhachev V. Lavroukov A. Kutepov S. Quarto R. Mastrogiacomo M. Cancedda R. (2007). Stem cells associated with macroporous bioceramics for long bone repair: 6- to 7-year outcome of a pilot clinical study. Tissue Eng. 13, 947–955 10.1089/ten.2006.0271 17484701

[B66] Menon L. G. Picinich S. Koneru R. Gao H. Lin S. Y. Koneru M. Mayer-Kuckuk P. Glod J. Banerjee D. (2007). Differential gene expression associated with migration of mesenchymal stem cells to conditioned medium from tumor cells or bone marrow cells. Stem Cells 25, 520–528 10.1634/stemcells.2006-0257 17053212

[B67] Morigi M. Imberti B. Zoja C. Corna D. Tomasoni S. Abbate M. Rottoli D. Angioletti S. Benigni A. Perico N. Alison M. Remuzzi G. (2004). Mesenchymal stem cells are renotropic, helping to repair the kidney and improve function in acute renal failure. J. Am. Soc. Nephrol. 15, 1794–1804 10.1097/01.ASN.0000128974.07460.34 15213267

[B68] Musina R. A. Belyavski A. V. Tarusova O. V. Solovyova E. V. Sukhikh G. T. (2008). Endometrial mesenchymal stem cells isolated from the menstrual blood. Bull. Exp. Biol. Med. 145, 539–543 19110612 10.1007/s10517-008-0136-0

[B69] Nilsson J. Skog J. Nordstrand A. Baranov V. Mincheva-Nilsson L. Breakefield X. O. Widmark A. (2009). Prostate cancer-derived urine exosomes: a novel approach to biomarkers for prostate cancer. Br. J. Cancer 100, 1603–1607 10.1038/sj.bjc.6605058 19401683 PMC2696767

[B70] Nishisho T. Hata K. Nakanishi M. Morita Y. Sun-Wada G. H. Wada Y. Yasui N. Yoneda T. (2011). The a3 isoform vacuolar type H^+^-ATPase promotes distant metastasis in the mouse B16 melanoma cells. Mol. Cancer Res. 9, 845–855 10.1158/1541-7786.MCR-10-0449 21669964

[B71] Ortiz L. A. Gambelli F. McBride C. Gaupp D. Baddoo M. Kaminski N. Phinney D. G. (2003). Mesenchymal stem cell engraftment in lung is enhanced in response to bleomycin exposure and ameliorates its fibrotic effects. Proc. Natl. Acad. Sci. U.S.A. 100, 8407–8411 10.1073/pnas.1432929100 12815096 PMC166242

[B72] Otsuru S. Gordon P. L. Shimono K. Jethva R. Marino R. Phillips C. L. Hofmann T. J. Veronesi E. Dominici M. Iwamoto M. Horwitz E. M. (2012). Transplanted bone marrow mononuclear cells and MSCs impart clinical benefit to children with osteogenesis imperfecta through different mechanisms. Blood. [Epub ahead of print]. 10.1182/blood-2011-12-400085 22829629 PMC3433095

[B73] Parekkadan B. van Poll D. Suganuma K. Carter E. A. Berthiaume F. Tilles A. W. Yarmush M. L. (2007). Mesenchymal stem cell-derived molecules reverse fulminant hepatic failure. PLoS ONE 2:e941 10.1371/journal.pone.0000941 17895982 PMC1978513

[B74] Parolini I. Federici C. Raggi C. Lugini L. Palleschi S. De Milito A. Coscia C. Iessi E. Logozzi M. Molinari A. Colone M. Tatti M. Sargiacomo M. Fais S. (2009). Microenvironmental pH is a key factor for exosome traffic in tumor cells. J. Biol. Chem. 284, 34211–34222 10.1074/jbc.M109.041152 19801663 PMC2797191

[B75] Pegtel D. M. Cosmopoulos K. Thorley-Lawson D. A. van Eijndhoven M. A. Hopmans E. S. Lindenberg J. L. de Gruijl T. D. Würdinger T. Middeldorp J. M. (2010). Functional delivery of viral miRNAs via exosomes. Proc. Natl. Acad. Sci. U.S.A. 107, 6328–6333 10.1073/pnas.0914843107 20304794 PMC2851954

[B76] Peinado H. Aleèkoviæ M. Lavotshkin S. Matei I. Costa-Silva B. Moreno-Bueno G. Hergueta-Redondo M. Williams C. García-Santos G. Ghajar C. M. Nitadori-Hoshino A. Hoffman C. Badal K. Garcia B. A. Callahan M. K. Yuan J. Martins V. R. Skog J. Kaplan R. N. Brady M. S. Wolchok J. D. Chapman P. B. Kang Y. Bromberg J. Lyden D. (2012). Melanoma exosomes educate bone marrow progenitor cells toward a pro-metastatic phenotype through MET. Nat. Med. 18, 883–891 10.1038/nm.2753 22635005 PMC3645291

[B77] Pittenger M. F. Mackay A. M. Beck S. C. Jaiswal R. K. Douglas R. Mosca J. D. Moorman M. A. Simonetti D. W. Craig S. Marshak D. R. (1999). Multilineage potential of adult human mesenchymal stem cells. Science 284, 143–147 10.1126/science.284.5411.143 10102814

[B78] Ponte A. L. Marais E. Gallay N. Langonne A. Delorme B. Herault O. Charbord P. Domenech J. (2007). The *in vitro* migration capacity of human bone marrow mesenchymal stem cells: comparison of chemokine and growth factor chemotactic activities. Stem Cells 25, 1737–1745 10.1634/stemcells.2007-0054 17395768

[B79] Rader D. J. Parmacek M. S. (2012). Secreted miRNAs suppress atherogenesis. Nat. Cell Biol. 14, 233–235 10.1038/ncb2452 22373869 PMC4111246

[B80] Rasmusson I. Le Blanc K. Sundberg B. Ringdén O. (2007). Mesenchymal stem cells stimulate antibody secretion in human B cells. Scand. J. Immunol. 65, 336–343 10.1111/j.1365-3083.2007.01905.x 17386024

[B81] Ratajczak J. Miekus K. Kucia M. Zhang J. Reca R. Dvorak P. Ratajczak M. Z. (2006). Embryonic stem cell-derived microvesicles reprogram hematopoietic progenitors: evidence for horizontal transfer of mRNA and protein delivery. Leukemia 20, 847–856 10.1038/sj.leu.2404132 16453000

[B82] Ries C. Egea V. Karow M. Kolb H. Jochum M. Neth P. (2007). MMP-2, MT1-MMP, and TIMP-2 are essential for the invasive capacity of human mesenchymal stem cells: differential regulation by inflammatory cytokines. Blood 109, 4055–4063 10.1182/blood-2006-10-051060 17197427

[B83] Roche S. D'Ippolito G. Gomez L. A. Bouckenooghe T. Lehmann S. Montero-Menei C. N. Schiller P. C. (2012). Comparative analysis of protein expression of three stem cell populations: models of cytokine delivery system *in vivo*. Int. J. Pharm. [Epub ahead of print]. 10.1016/j.ijpharm.2011.12.041 22285475

[B84] Rojas M. Xu J. Woods C. R. Mora A. L. Spears W. Roman J. Brigham K. L. (2005). Bone marrow-derived mesenchymal stem cells in repair of the injured lung. Am. J. Respir. Cell Mol. Biol. 33, 145–152 10.1165/rcmb.2004-0330OC 15891110 PMC2715309

[B85] Roorda B. D. ter Elst A. Kamps W. A. de Bont E. S. (2009). Bone marrow-derived cells and tumor growth: contribution of bone marrow-derived cells to tumor micro-environments with special focus on mesenchymal stem cells. Crit. Rev. Oncol. Hematol. 69, 187–198 10.1016/j.critrevonc.2008.06.004 18675551

[B86] Rosario C. M. Yandava B. D. Kosaras B. Zurakowski D. Sidman R. L. Snyder E. Y. (1997). Differentiation of engrafted multipotent neural progenitors towards replacement of missing granule neurons in meander tail cerebellum may help determine the locus of mutant gene action. Development 124, 4213–4224 9334270 10.1242/dev.124.21.4213

[B88] Rubio D. Garcia S. Paz M. F. De la Cueva T. Lopez-Fernandez L. A. Lloyd A. C. Garcia-Castro J. Bernad A. (2008). Molecular characterization of spontaneous mesenchymal stem cell transformation. PLoS ONE 3:e1398 10.1371/journal.pone.0001398 18167557 PMC2151133

[B89] Sasportas L. S. Kasmieh R. Wakimoto H. Hingtgen S. van de Water J. A. Mohapatra G. Figueiredo J. L. Martuza R. L. Weissleder R. Shah K. (2009). Assessment of therapeutic efficacy and fate of engineered human mesenchymal stem cells for cancer therapy. Proc. Natl. Acad. Sci. U.S.A. 106, 4822–4827 10.1073/pnas.0806647106 19264968 PMC2660771

[B90] Semedo P. Palasio C. G. Oliveira C. D. Feitoza C. Q. Gonçalves G. M. Cenedeze M. A. Wang P. M. Teixeira V. P. Reis M. A. Pacheco-Silva A. Câmara N. O. (2009). Early modulation of inflammation by mesenchymal stem cell after acute kidney injury. Int. Immunopharmacol. 9, 677–682 10.1016/j.intimp.2008.12.008 19146993

[B91] Schreml S. Babilas P. Fruth S. Orsó E. Schmitz G. Mueller M. B. Nerlich M. Prantl L. (2009). Harvesting human adipose tissue-derived adult stem cells: resection versus liposuction. Cytotherapy 11, 947–957 10.3109/14653240903204322 19903106

[B92] Schrepfer S. Deuse T. Reichenspurner H. Fischbein M. P. Robbins R. C. Pelletier M. P. (2007). Stem cell transplantation: the lung barrier. Transplant. Proc. 39, 573–576 10.1016/j.transproceed.2006.12.019 17362785

[B93] Sheng H. Wang Y. Jin Y. Zhang Q. Zhang Y. Wang L. Shen B. Yin S. Liu W. Cui L. Li N. (2008). A critical role of IFNgamma in priming MSC-mediated suppression of T cell proliferation through up-regulation of B7-H1. Cell Res. 18, 846–857 10.1038/cr.2008.80 18607390

[B94] Simon S. Roy D. Schindler M. (1993). Intracellular pH and the control of multidrug resistance. Proc. Natl. Acad. Sci. U.S.A. 91, 1128–1132 8302842 10.1073/pnas.91.3.1128 PMC521467

[B95] Skog J. Würdinger T. van Rijn S. Meijer D. H. Gainche L. Sena-Esteves M. Curry W. T. Jr. Carter B. S. Krichevsky A. M. Breakefield X. O. (2008). Glioblastoma microvesicles transport RNA and proteins that promote tumour growth and provide diagnostic biomarkers. Nat. Cell Biol. 10, 1470–1476 10.1038/ncb1800 19011622 PMC3423894

[B96a] Snykers S. De Kock J. Tamara V. Rogiers V. (2011). Hepatic differentiation of mesenchymal stem cells: *in vitro* strategies. Methods Mol. Biol. 698, 305–314 10.1007/978-1-60761-999-4_23 21431528

[B98] Stagg J. Pommey S. Eliopoulos N. Galipeau J. (2006). Interferon-gamma-stimulated mar-row stromal cells: a new type of nonhematopoietic antigen-presenting cell. Blood 107, 2570–2577 10.1182/blood-2005-07-2793 16293599

[B99] Strassburg S. Hodson N. W. Hill P. I. Richardson S. M. Hoyland J. A. (2012). Bi-directional exchange of membrane components occurs during co-culture of mesenchymal stem cells and nucleus pulposus cells. PLoS ONE 7:e33739 10.1371/journal.pone.0033739 22438989 PMC3305345

[B100] Théry C. Amigorena S. Raposo G. Clayton A. (2006). Isolation and characterization of exosomes from cell culture supernatants and biological fluids. Curr. Protoc. Cell Biol. Chapter 3, Unit 3.22. 10.1002/0471143030.cb0322s30 18228490

[B101] Till J. E. McCulloch E. A. (1964). Repair processes in irradiated mouse hematopoietic tissue. Ann. N.Y. Acad. Sci. 114, 115–125 10.1111/j.1749-6632.1964.tb53566.x 14125958

[B102] Tögel F. Hu Z. Weiss K. Isaac J. Lange C. Westenfelder C. (2005). Administered mesenchymal stem cells protect against ischemic acute renal failure through differentiation-independent mechanisms. Am. J. Physiol. Renal Physiol. 289, F31–F42 10.1152/ajprenal.00007.2005 15713913

[B103] Toma C. Pittenger M. F. Cahill K. S. Byrne B. J. Kessler P. D. (2002). Human mesenchymal stem cells differentiate to a cardiomyocyte phenotype in the adult murine heart. Circulation 105, 93–98 10.1161/hc0102.101442 11772882

[B104] Tondreau T. Dejeneffe M. Meuleman N. Stamatopoulos B. Delforge A. Martiat P. Bron D. Lagneaux L. (2008). Gene expression pattern of functional neuronal cells derived from human bone marrow mesenchymal stromal cells. BMC Genomics 9, 166 10.1186/1471-2164-9-166 18405367 PMC2358905

[B97] Trzaska K. A. Kuzhikandathil E. V. Rameshwar P. (2007). Specification of a dopaminergic phenotype from adult human mesenchymal stem cells. Stem Cells 25, 2797–2808 10.1634/stemcells.2007-0212 17656644

[B105] Trzaska K. A. Rameshwar P. (2011). Dopaminergic neuronal differentiation protocol for human mesenchymal stem cells. Methods Mol. Biol. 698, 295–303 10.1007/978-1-60761-999-4_22 21431527

[B106] Valadi H. Ekström K. Bossios A. Sjöstrand M. Lee J. J. Lötvall J. O. (2007). Exosome-mediated transfer of mRNAs and microRNAs is a novel mechanism of genetic exchange between cells. Nat. Cell Biol. 9, 654–659 10.1038/ncb1596 17486113

[B107] Valenick L. V. Hsia H. C. Schwarzbauer J. E. (2005). Fibronectin fragmentation promotes alpha4beta1 integrin-mediated contraction of a fibrin-fibronectin provisional matrix. Exp. Cell Res. 309, 48–55 10.1016/j.yexcr.2005.05.024 15992798

[B108] Verweij F. J. van Eijndhoven M. A. Hopmans E. S. Vendrig T. Wurdinger T. Cahir-McFarland E. Kieff E. Geerts D. van der Kant R. Neefjes J. Middeldorp J. M. Pegtel D. M. (2011). LMP1 association with CD63 in endosomes and secretion via exosomes limits constitutive NF-κ B activation. EMBO J. 30, 2115–2129 10.1038/emboj.2011.123 21527913 PMC3117644

[B109] Voermans C. Gerritsen W. R. von dem Borne A. E. van der Schoot C. E. (1999). Increased migration of cord blood-derived CD34 + cells, as compared to bone marrow and mobilized peripheral blood CD34 + cells across uncoated or fibronectin-coated filters. Exp. Hematol. 27, 1806–1814 10641598 10.1016/s0301-472x(99)00113-7

[B110] Wang J. Guan E. Roderiquez G. Calvert V. Alvarez R. Norcross M. A. (2001). Role of tyrosine phosphorylation in ligand-independent sequestration of CXCR4 in human primary monocytes-macrophages. J. Biol. Chem. 276, 49236–49243 10.1074/jbc.M108523200 11668182

[B111] Wang T. Xu Z. Jiang W. Ma A. (2006). Cell-to-cell contact induces mesenchymal stem cell to differentiate into cardiomyocyte and smooth muscle cell. Int. J. Cardiol. 109, 74–81 10.1016/j.ijcard.2005.05.072 16122823

[B112] Warnke P. H. Springer I. N. Wiltfang J. Acil Y. Eufinger H. Wehmöller M. Russo P. A. Bolte H. Sherry E. Behrens E. Terheyden H. (2004). Growth and transplantation of a custom vascularised bone graft in a man. Lancet 364, 766–770 10.1016/S0140-6736(04)16935-3 15337402

[B113] Wynn R. F. Hart C. A. Corradi-Perini C. O'Neill L. Evans C. A. Wraith J. E. Fairbairn L. J. Bellantuono I. (2004). A small proportion of mesenchymal stem cells strongly expresses functionally active CXCR4 receptor capable of promoting migration to bone marrow. Blood 104, 2643–2645 10.1182/blood-2004-02-0526 15251986

[B114] Xu X. Zhang X. Wang S. Qian H. Zhu W. Cao H. Wang M. Chen Y. Xu W. (2011). Isolation and comparison of mesenchymal stem-like cells from human gastric cancer and adjacent non-cancerous tissues. J. Cancer Res. Clin. Oncol. 137, 495–504 10.1007/s00432-010-0908-6 20473524 PMC11827778

[B116] Yang B. Wu X. Mao Y. Bao W. Gao L. Zhou P. Xie R. Zhou L. Zhu J. (2009). Dual-targeted antitumor effects against brainstem glioma by intravenous delivery of tumor necrosis factor-related, apoptosis-inducing, ligand-engineered human mesenchymal stem cells. Neurosurgery 65, 610–624 10.1227/01.NEU.0000350227.61132.A7 19687708

[B117] Zhang Z. L. Tong J. Lu R. N. Scutt A. M. Goltzman D. Miao D. S. (2009). Therapeutic potential of non-adherent BM-derived mesenchymal stem cells in tissue regeneration. Bone Marrow Transplant. 43, 69–81 10.1038/bmt.2008.260 18711348

[B118] Zhu W. Huang L. Li Y. Zhang X. Gu J. Yan Y. Xu X. Wang M. Qian H. Xu W. (2012). Exosomes derived from human bone marrow mesenchymal stem cells promote tumor growth *in vivo*. Cancer Lett. 315, 28–37 10.1016/j.canlet.2011.10.002 22055459

[B119] Zvaifler N. J. Marinova-Mutafchieva L. Adams G. Edwards C. J. Moss J. Burger J. A. Maini R. N. (2000). Mesenchymal precursor cells in the blood of normal individuals. Arthritis Res. 2, 477–488 10.1186/ar130 11056678 PMC17820

